# Challenges facing professional nurses implementing the Integrated Management of Childhood Illness programme in rural primary health care clinics, Limpopo Province, South Africa

**DOI:** 10.4102/safp.v62i1.5060

**Published:** 2020-05-25

**Authors:** Livhuwani Tshivhase, Mankuku M. Madumo, Indiran Govender

**Affiliations:** 1 Department of Nursing, Faculty of Health Science, Sefako Makgatho Health Sciences University, Pretoria, South Africa; 2 Department of Family Medicine and Primary Health Care, University of Pretoria, Pretoria, South Africa

**Keywords:** challenges, professional nurses, Integrated Management of Childhood Illness, implementation, primary health care

## Abstract

**Background:**

Under-five mortality and morbidity could be reduced through increased implementation of the Integrated Management of Childhood Illness (IMCI) strategy. The aim of the study was to determine challenges facing IMCI-trained professional nurses on implementing this strategy when managing children less than 5 years of age.

**Methods:**

A quantitative descriptive survey method was used. The target populations were IMCI-trained professional nurses with the sample of 208 respondents. Data were collected through self-report questionnaires and analysed using statistical analysis system software.

**Results:**

The implementation of the IMCI strategy by IMCI-trained professional nurses in Vhembe primary health care (PHC) clinics continues to face challenges, making it difficult for professional nurses to follow guidelines. These challenges range from staff barriers, management barriers, poor management process and poor infrastructure. All these challenges lead to poor-quality under-five patient care.

**Conclusion:**

Regardless of the IMCI strategy being implemented since its inception in 1999, the under-five mortality remains not reduced. This is related to the identified challenges facing the IMCI-trained professional nurses implementing the strategy.

## Introduction

Every year in low-income countries, more than 10.2 million children die before they reach their fifth birthday, most of them in their first year of life.^1,2^ The highest death rates, of one child in 13 dying before his or her fifth birthday, are found in sub-Saharan Africa where child mortality is increasing.^3^

In 2018, the World Health Organization (WHO) reported that pneumonia, diarrhoea and malaria were among the highest common causes of under-five child mortality. Nutrition-related factors were also alarming as causing 45% of death to the under-five in sub-Saharan countries. These are priority illnesses that are addressed by the WHO’s Integrated Management of Childhood Illness (IMCI) strategy and the United Nations Children’s Fund (UNICEF).^4^ If IMCI strategy is well implemented, some of the diseases such as diarrhoea and pneumonia could have been prevented through vaccination such as rotavirus and pneumococcal vaccines.

Even diseases such as malaria would not be easily missed as all under-five children who present with fever are screened and tested for it. In IMCI management, there are six steps, namely: Step 1: Assessment; Step 2: Classify; Step 3: Identify treatment; Step 4: Treat; Step 5: Counsel and Step 6: Follow-up.^5^ Step 5 of the IMCI strategy includes a process of counselling the mother and/or caregiver of every child ensuring that all identified conditions are continuously monitored at home. Integrated Management of Childhood Illness strategy recommends that IMCI-trained professional nurses and doctors should check the caregiver’s or parent’s understanding of the advice given and show them the method of administering the first treatment dose to ensure that the child receives adequate care at home.^5^ If optimally implemented, the IMCI strategy could assist in the reduction of under-five deaths, and most countries could attain the sustainable development goal (SDG) number three: good health and well-being, which includes reducing child mortality^5^ by the year 2030. Through optimal use of the IMCI strategy, danger signs such as lethargy are identified, as well as obtaining a history such as the child’s ability to drink, breastfeed, degree of vomiting and the presence of convulsions or unconsciousness.

In Botswana, the IMCI strategy implementation led to the decrease in under-five child mortality from 90/1000 in 2000 to 48/1000 in 2010.^2^ Despite the inception of IMCI strategy in 1996, South Africa remained challenged with under-five child mortality that was estimated at 45.1% death per 1000 births in 2015.^6^

After 13 years, the implementation of the IMCI strategy was reported to be poor, not only in South Africa but also globally.^7,8,9,10,11^ The 2nd Triennial Report on Morbidity and Mortality^12^ in the Limpopo Province indicated that 95% of primary health care (PHC) nurses were IMCI-trained in 2014, but under-five child mortality in 2015 in that province was the highest in the country.^11^ It seems that the availability of IMCI-trained professional nurses does not always translate to adequate IMCI strategy implementation. Under-five children are continuing to die with treatable and preventable illnesses that could have been addressed through implementation of the IMCI strategy.

There is sufficient evidence of good quality care outcomes from IMCI strategy, and it is therefore of vital importance to focus on what could be performed to optimise the implementation of this strategy.^13^ This study is therefore focused on determining the challenges of IMCI-trained professional nurses in implementing the IMCI strategy at the PHC clinics of Vhembe district.

## Methods

A quantitative descriptive survey was used for this study. The data were collected from January to March 2017. The population was composed of IMCI-trained professional nurses who were working in the PHC clinics of Vhembe district. Vhembe district is the northern largest most rural part of Limpopo Province which shares its borders with Zimbabwe. The sample calculator was used to determine the number of respondents to participate in this study. Systematic random sampling was performed in 52 PHC clinics, and an additional 20 PHC clinics were conveniently sampled after encountering floods challenges. However, all the respondents met the inclusion criteria. A total of 208 respondents were enrolled. Data were collected through self-administered questionnaires which the respondents completed at their work stations. The questionnaire had the following sections: demographic as well as professional data; challenges facing IMCI-trained professional nurses in implementing the IMCI strategy; perceived challenges of IMCI-trained professional nurses on IMCI strategy implementation; and recommendations for improving IMCI strategy implementation. Data were analysed using statistical analysis system (SAS) (SAS Institute Inc., Carey, North Carolina, United States). Experts in child health and a qualified statistician were consulted during the research planning and data analysis phases. The questionnaire was modified from a study conducted in Botswana^16^ for which the author’s permission was obtained. A pilot study was conducted to test the questionnaire, and two items were modified to improve meaning as the pilot respondents left the space blank.

## Results

Two-hundred and eight respondents participated in this study. The minimum age for respondents was 24 years, and the maximum age was 63 years. The majority of the study respondents were women (90.4%).

All professional nurses were IMCI-trained but differed with other skills, for example, midwifery (72%), psychiatric (38%), general nursing (only 54%) and community nursing (52%). Sixty per cent of the respondents had a Health Assessment Treatment and Care (PHC) Diploma.

A majority of the respondents (51%) were trained in IMCI between 2006 and 2012. Many of the respondents (37%) have been managing under-five children for more than 10 years. More than half of all the respondents (73%) responded that they had never been followed up post-IMCI training, as shown in Table 2. The majority of the respondents (74%) had undergone in-service training on IMCI and 26% had received pre-service training.

**TABLE 1:** Demographic profiles of respondents (*n* = 208).

Variable	Number	%
**Gender**		
Female	188	90
Male	20	10
**Qualifications Professional nurse**		
Degree in nursing	84	41
Diploma in nursing	111	53
Degree and diploma in nursing	13	6
**Years of experience after IMCI training**		
3–5 years	29	14
5–10 years	107	51
Above 10 years	72	35
**Age distribution of respondents**		
Number	208	100
Mean age	46.4	Standard dev ± 8.6
Min or Max	24/63	-

IMCI, Integrated Management of Childhood Illness; Min, minimum; Max, maximum.

**TABLE 2:** Challenges facing Integrated Management of Childhood Illness–trained professional nurses implementing the Integrated Management of Childhood Illness strategy.

Statement	Frequency (*n*)	%
**1. IMCI has boosted my confidence and skills in managing under-five children**		
Agree	202	97
Disagree	5	2
Neutral	1	1
**2. IMCI has led to longer waiting times because of time spent applying IMCI stages**		
Agree	140	67
Disagree	46	22
Neutral	22	11
**3. IMCI is partially implemented as some children are treated by non-IMCI-trained nurses**		
Agree	115	55
Disagree	67	32
Neutral	26	13
**4. IMCI has reduced follow-up because of thorough and accurate case management at initial visit**		
Agree	139	67
Disagree	50	24
Neutral	19	9
**5. It is not practical to always refer to IMCI chart booklet during every case management**		
Agree	90	43
Disagree	103	50
Neutral	15	7
**6. All IMCI-trained nurses at our clinic apply all stages of IMCI protocol**		
Agree	101	49
Disagree	63	30
Neutral	44	21
**7. Our facility layout does not make it easy to practise IMCI**		
Agree	69	33
Disagree	117	56
Neutral	22	11
**8. Case management practices of IMCI-trained and non-IMCI-trained nurses are inconsistent**		
Agree	61	29
Disagree	112	54
Neutral	35	17

IMCI, Integrated Management of Childhood Illness.

A majority of the respondents (62%) had undergone the 11-day IMCI training and 38% underwent pre-service training. Table 1 displays the demographics of the respondents. Tables 2 to 4 illustrate the challenges facing IMCI-trained professional nurses implementing the IMCI strategy. Table 3 shows the challenges in relation to time used to consult under-five children when using IMCI strategy. Table 5 presents the recommendations to improve IMCI strategy implementation.

**TABLE 3:** Time spent on clients with and without using Integrated Management of Childhood Illness guidelines.

Response	Using IMCI guidelines consultation is quicker		Not using IMCI guidelines consultation is quicker		Value
	*N*	%	*N*	%	
Agree	40	19	102	49	< 0.001†
Disagree	157	76	91	44	< 0.001†
Neutral	11	5	15	7	0.544
**Total**	**208**	**100**	**208**	**100**	**-**

IMCI, Integrated Management of Childhood Illness.

† , The two percentages differ significantly (Fisher’s exact test).

**TABLE 4:** Challenges facing Integrated Management of Childhood Illness–trained professional nurses implementing the Integrated Management of Childhood Illness strategy.

Statement	Frequency	%
**1. IMCI is a user-friendly strategy for health workers**		
Agree	192	92
Disagree	10	5
Neutral	6	3
**2. IMCI strategy is easy to understand and apply**		
Agree	187	90
Disagree	12	6
Neutral	9	4
**3. IMCI protocol is too long**		
Agree	92	44
Disagree	95	46
Neutral	21	10
**4. IMCI is tedious or tiresome or boring**		
Agree	66	32
Disagree	83	40
Neutral	59	28
**5. IMCI is time-consuming**		
Agree	98	47
Disagree	90	43
Neutral	20	10
**6. IMCI is not practical to use at facility level**		
Agree	35	17
Disagree	155	74
Neutral	18	9
**7. IMCI is difficult to understand and apply**		
Agree	19	9
Disagree	179	86
Neutral	10	5
**8. Supervisors do not understand the rationale of IMCI strategy**		
Agree	13	6
Disagree	178	86
Neutral	17	8
**9. My supervisor is not IMCI-trained**		
Agree	23	11
Disagree	165	79
Neutral	20	10
**10. Patient–nurse ratio does not allow the use of the IMCI strategy**		
Agree	77	37
Disagree	113	54
Neutral	18	9
**11. IMCI guidelines are too simplistic; undermine my clinical training**		
Agree	26	13
Disagree	169	81
Neutral	13	6.
**12. IMCI drugs are frequently out of stock**		
Agree	36	17
Disagree	143	69
Neutral	29	14
**13. Some clinical officers and doctors have negative attitudes towards IMCI**		
Agree	71	34
Disagree	109	52
Neutral	28	14
**14. IMCI wall charts and chart booklets are frequently unavailable**		
Agree	69	33
Disagree	128	62
Neutral	11	5
**15. Lack of IMCI follow-up by IMCI facilitators**		
Agree	152	73
Disagree	44	21
Neutral	12	6
**16. Lack of supervision by IMCI trainers**		
Agree	144	69
Disagree	52	25
Neutral	12	6
**17. Health facility not fully equipped to support use of IMCI strategy procedures**		
Agree	75	36
Disagree	103	50
Neutral	30	14.

IMCI, Integrated Management of Childhood Illness.

**TABLE 5:** Recommendations to improve integrated management of childhood illness strategy implementation.

No.	Recommendations	Agree (%)	Disagree (%)	Neutral (%)
1.	Integration of IMCI into pre-service training	84	8	8
2.	Scaling up of IMCI into pre-service training	89	6	5
3.	Extending IMCI training to lower cadres of nurses in relation to their scope of practice, for example, auxiliary and enrolled nurse	80	11	9
4.	Extension of IMCI training to senior manager	93	3	4
5.	All nurses tending under-fives should be IMCI trained	94	4	2
6.	IMCI used as a criterion for daily allocation of nurses	61	26	13
7.	Provide facility layout that allows practice of all IMCI skills	89	5	6
8.	Ensure availability of IMCI drugs, wall charts, booklet at all times	94	4	2
9.	Address problem of understaffing	92	5	3
10.	Follow-up and supervision of IMCI-trained nurses	92	4	4
11.	IMCI national focal person should advocate for governmental policy support	91	3	6
12.	IMCI national focal person should advocate for more resources from health planners	92	3	5

IMCI, Integrated Management of Childhood Illness.

## Discussion

Respondents for this study included 208 IMCI-trained professional nurses working in the PHC clinics. Majority of the respondents were female (90%), and this could have been because of the fact that nursing in South Africa is a predominantly female profession.^14^ All the respondents were more than 5-year post-IMCI training and have been practising for more than 2 years, which implies that they are experienced in IMCI implementation and were expected to follow the IMCI guidelines.

## Challenges facing Integrated Management of Childhood Illness-trained professional nurses implementing the Integrated Management of Childhood Illness strategy

A significant greater number of nurses (67%) reported that using the IMCI guidelines during their consultations took more time than when they were not using them. This finding is similar to those of previous studies that reported consultations to be more time-consuming when implementing the IMCI strategy as compared to traditional consultations.^15^ Traditional consultations are those consultations in which doctors and nurses consult under-five children without referring to the IMCI guidelines and respondents referred to them as quicker. The time-consuming aspect of IMCI in comparison with traditional consultations was reported by Horwood et al.^17^ Maleshane^18^ reported that in North West Province using the IMCI strategy took between 30 min and 45 min per patient. In contrast, Kruger, Heinzel-Gutenbrunner and Ali^19^ reported that health workers used 7–12 min to perform a complete assessment of under-five children.

Many (55%) respondents indicated that IMCI is only partially implemented as other children are treated by non-IMCI-trained professional nurses in their facilities. There is a plea from PHC clinics for trained IMCI professional nurses as indicated by 92% of the respondents. The same findings that indicated a lack of IMCI-trained staff were also found in other countries.^8,17^

A majority of respondents (67%) believed that the IMCI strategy reduced the number of follow-up visits through accurate case management and that positive outcomes of the IMCI strategy were evident. Mupara^16^ supported the findings by stating that those who had seen for themselves the benefits of the IMCI strategy were eager to implement the strategy irrespective of its challenges. On the contrary, Lange et al.^11^ alluded to the fact that health workers seemed unconvinced that the IMCI strategy was valuable in managing under-five children and that this was a major reason for poor adherence to the IMCI strategy.

Half of this study’s respondents (50%) believed that it is not always practical to refer to IMCI guidelines when treating children below 5 years of age. This statement elicited mixed feelings among respondents. It was therefore important to further discover the challenges in the use of the IMCI guidelines.

Only 49% of respondents stated that all IMCI-trained professional nurses apply all stages of the IMCI guidelines. This statement indicates the limited application of the IMCI strategy by trained staff. This response was also reported in a study conducted in Indonesia including Uganda and Kenya that at times nurses did not fully assess or classify the child they were managing according to the IMCI guidelines.^19^

Thirty-three per cent of the respondents stated that their facility’s layout did not make it easy to apply IMCI steps, such as observing the first treatment intake. The facility had limited space, and the respondents indicated that this was a barrier to IMCI implementation. It was also recommended by 89% of the study’s respondents that facility layout should be improved to promote IMCI strategy implementation. Several studies have also reported that a poor infrastructure is not conducive to IMCI implementation.^7,10^

Despite the challenges experienced by IMCI-trained professional nurses, majority of the respondents (97%) also believed that IMCI boosted their confidence in skills and managing the under-five children. The same findings were cited in a study by Adekanye and Odetola,^20^ indicating that the child illness management skills of health workers had improved. Kiplagat et al.^10^ confirmed that health workers who used IMCI guidelines were able to classify and treat children even without a laboratory investigation.

Other challenges experienced included the question whether IMCI was regarded as too simplistic, and if it undermined their clinical skills, the majority (81%) responded in the negative manner, implying that IMCI was regarded as a necessary skill needed by professional nurses. The results concur with those of Mupara^16^ who reported that health workers trained in IMCI never perceived it as too simplistic and less effective than their clinical training.

Doctors are also implementing IMCI in South Africa. The study results also proved that doctors have positive attitude towards the strategy implementation as confirmed by 52% of respondents. This result is inconsistent with the study conducted in Kenya where participants of the study believed that doctors had negative attitudes towards IMCI implementation.^9,20,21,22^

In contrast to the 44% of the respondents who agreed that the IMCI protocol is too long and time-consuming, 92% of the same respondents indicated that IMCI is user-friendly and a further 90% believed that IMCI strategy is easy to understand and apply. The study conducted by Mugala et al.^23^ in Zambia concurs with the findings of this study in that the consultation was found to be time-consuming. In the same manner, the findings of the study in Botswana never found the IMCI strategy difficult to apply.^16^

Supervisors are the integral part of IMCI implementation; therefore, they need to be IMCI-trained to understand the strategy. Eight-nine per cent of the respondents in the study indicated that their supervisor understood the rationale for using the IMCI strategy. However, the results also revealed that some supervisors were not IMCI-trained as indicated by 11% of respondents. A further 69% of respondents indicated that they lacked supervision of IMCI strategy implementation at their workplace after they were trained. It is therefore imperative to advocate for the IMCI training of clinic supervisors so that they can promote the IMCI strategy implementation and be able to supervise staff post-training. The finding is similar to that of several studies reporting national assessment surveys in Namibia, Kenya, Tanzania, Uganda and South Africa in which implementers of IMCI strategy also indicated that they lacked supervision.^9,10, 19,24^

World Health Organization^5^ recommends that trainees be followed up within 4–6 weeks, but in this study, 73% of the respondents indicated that they did not have follow-up after their training. Follow-up is important as it is associated with reinforcing the learned skills. This may mean that nurses trained are not competent in implementing IMCI as their skills were not reinforced through follow-up after training. This lack of follow-up was also reported in Botswana and Kenya.^16,20^

Resources are vital for adequate IMCI implementation. Although 62% of respondents indicated that the IMCI wall charts, chart booklet and IMCI drugs were available, 94% of the same respondents recommended that chart booklets should be available at the clinic for promoting the IMCI strategy. This finding may imply that these were unavailable at some centres and respondents could have been afraid to indicate their unavailability. The results of Titaley et al.^22^ were similar in that there were shortages of IMCI wall charts and chart booklets in health centres that made the implementation of IMCI inadequate. Several studies of the IMCI programme had also indicated a lack of specific drugs for IMCI as a barrier to its implementation.^10,24,25^

Human resource is required for the implementation of IMCI strategy; however, 46% of the respondents indicated that they had challenges with the patient–nurse ratio thereby creating a barrier to IMCI implementation. Surprisingly, a larger proportion (92%) suggested improving the patient–nurse ratio to promote IMCI implementation as the study recommendations. Goga and Muhe^21^ support these results, arguing that staff shortages are a barrier to successful IMCI implementation.

Shortages of staff trained in the strategy lead to increased workload and thus compromise IMCI implementation. The provision of resources in PHC clinics, as well as conducting in-service training and supervising IMCI-trained staff, could promote the quality of care for under-five children through an improved IMCI implementation.

## Recommendations

Measures that could be considered to address the desire of these nurses for improved IMCI training at all nursing levels include scaling up of IMCI training on the job. The extension of IMCI training should be made to lower cadres of nurses as they are the first contact of under-five children and could thus identify problems early and refer appropriately. In addition, senior nurse managers should be trained as they could help in the supervision and support of those implementing the programme.

Structural elements such as IMCI drugs should be prioritised by management as very important so that children less than 5 years old are treated appropriately using the prescribed drugs. Infrastructural budgets from the National Department of Health (NDoH) should be prioritised so that PHC clinics have adequate consulting rooms for IMCI services.

Processes such as supervision, in-service training and follow-up after training should be the responsibility of a district IMCI manager with a schedule honoured by all medical personnel.

## Conclusion

Integrated Management of Childhood Illness–trained professional nurses find it difficult to implement the IMCI strategy despite their knowledge and acknowledgment of its benefits. Challenges that were shared included the fact that structural elements such as inadequate infrastructure, lack of IMCI drugs, shortages of material resources as well as human resources have a compromising effect on the quality of care rendered to under-five children. Process elements that are not conducted, such as in-service training, supportive supervision and follow-up after IMCI training, were reported to hamper IMCI strategy implementation by the majority of these respondents.

## References

[1] Jibo A, Iliayasu Z, Abubakar I, Umar L, Hassan A. Community-integrated management of childhood illnesses (C-IMCI) and key household practices in Kano, North West Nigeria. Sub-Saharan Afr J Med. 2014;1(2):70–77. 10.4103/2384-5147.136810 PMC10767726 38188281

[2] Nkosi Z, Botshabelo R, Jorosi H, Makole N, Nkomo G, Ruele S. The implementation of the integrated management of childhood illness (IMCI) strategy guidelines in Botswana. Afr J Nurs Midwifery. 2012;14(2):90–103.

[3] Kalu N, Lufesi N, Havens D, Mortimer K. Implementation of World Health Organization integrated management of childhood illnesses (IMCI) guidelines for the assessment of pneumonia in the under 5s in Rural Malawi. PLoS One. 2016;11(5):e0155830. 10.1371/journal.pone.0155830 27187773 PMC4871345

[4] Maternal, newborn, child and adolescent health (IMCI) [homepage on the Internet]. c2013 [cited 2016 Apr 30]. Available from: https://www.who.int/maternalchildadolescent/topic/child/imci/en/

[5] IMCI chart booklet [homepage on the Internet]. c2014 [cited 2019 Nov 30]. Available from: https://www.who.int/maternalchildadolescent/topic/child/imci/en/

[6] South African statistic [homepage on the Internet]. c2015 [cited 2016 Sep 22]. Available from: https://www.southafrica.info/about/health/infant-mortality-240216.htm

[7] Mulaudzi M. Adherence to case management guidelines of integrated management of childhood illness (IMCI) by healthcare workers in Tshwane, South Africa. S Afr J Child Health. 2015;9(3):89. 10.7196/SAJCH.7959

[8] Vhuromu E, Davhana-Maselesele M. Experiences of primary health care nurses in implementing integrated management of childhood illnesses strategy at selected clinics of Limpopo Province. Curationis. 2009;32(3):a1224. 10.4102/curationis.v32i3.1224 20225745

[9] Pandya H, Slemming W, Saloojee H. Health system factors affecting implementation of integrated management of childhood illness (IMCI): Qualitative insight from a South African province. Health Policy Plan. 2018;33(2):171–182. 10.1093/heapol/czx154 29161375

[10] Kiplagat A, Musto R, Mwizamholya D, Morona D. Factors influencing the implementation of integrated management of childhood illness (IMCI) by healthcare workers at public health centres and dispensaries in Mwanza, Tanzania. BMC Public Health. 2014;14(1):277. 10.1186/1471-2458-14-277 24666561 PMC3987128

[11] Lange S, Mwisongo A, Mæstad O. Why don’t clinicians adhere more consistently to guidelines for the integrated management of childhood illness (IMCI)? Soc Sci Med. 2014;104:56–63. 10.1016/j.socscimed.2013.12.020 24581062

[12] KwaZulu – Natal Department of Health. Second Triennial Report on Mortality and Morbidity [Internet]. Kznhealth.gov.za: South Africa; 2014 [cited 2019 Nov 13]. Available from: http://www.kznhealth.gov.za/mcwh/2nd-CoMMiC-Triennial-Report-Abridged.pdf

[13] Meno FO, Makhado L, Matsipane M. Factors inhibiting implementation of integrated management of childhood illnesses(IMCI) in primary health care (PHC) facilities in Mafikeng sub-district. International Journal of Africa Nursing Sciences. 2019;11:1–7. 10.1016/j.ijans.2019.100161

[14] South African Nursing Council (SANC). *Nursing Act* (Act No. 33 of 2005) as amended. Pretoria: SANC; 2005.

[15] Buthelezi JKA. Implementation of customer care at the casualty Department of Edenvale Regional Hospital in Gauteng Province [unpublished thesis submitted in accordance with the requirement for the degree of Master of Public Administration]. Pretoria: University of South Africa; 2017.

[16] Mupara LM. Challenges identified by experienced IMCI-trained registered nurses in implementation of the IMCI strategy in Gaborone, Botswana [master’s dissertation]. Pretoria: University of South Africa; 2012.

[17] Horwood C, Vermaak K, Rollins N, Haskins L, Nkosi P, Qazi S. An evaluation of the quality of IMCI assessments among IMCI-trained health workers in South Africa. PLoS One. 2009;4(6):e5937. 10.1371/journal.pone.0005937 19536288 PMC2693922

[18] Maleshane MMY. Challenges of nurses in primary health setting regarding implementation of IMCI [master’s dissertation]. North West Province: North West University; 2012.

[19] Krüger C, Heinzel-Gutenbrunner M, Ali M. Adherence to the integrated management of childhood illness guidelines in Namibia, Kenya, Tanzania and Uganda: Evidence from the national service provision assessment surveys. BMC Health Serv Res. 2017;17(1):822. 10.1186/s12913-017-2781-3 29237494 PMC5729502

[20] Adekanye RNO, Odetola RNT. Awareness and implementation of integrated management of childhood illness (IMCI) among nurses in paediatric settings of selected hospitals in Ibadan, Nigeria. IOSR J Nurs Health Sci. 2014;3(5):29–34. 10.9790/1959-03532934

[21] Goga A, Muhe L. Global challenges with scale-up of the integrated management of childhood illness strategy: Results of a multi-country survey. BMC Public Health. 2011;11(1):503. 10.1186/1471-2458-11-503 21708029 PMC3155839

[22] Titaley C, Jusril H, Ariawan I, Soeharno N, Setiawan T, Weber M. Challenges to the implementation of the integrated management of childhood illness (IMCI) at community health centres in West Java province, Indonesia. WHO South-East Asia J Public Health. 2014;3(2):161. 10.4103/2224-3151.206732 28607302

[23] Mugala N, Mutale W, Kalesha P, Sinyinza E. Barriers to implementation of the HIV guidelines in the IMCI algorithm among IMCI-trained health workers in Zambia. BMC Pediatr. 2010;10(1). 10.1186/1471-2431-10-93 PMC3023733 21167016

[24] Mullei K, Wafula F, Goodman C. A case study of IMCI implementation in Kenya [homepage on the Internet]. c2008 [cited 2019 Nov 14]. Available from: https://www.researchgate.net/publication/238709033_A_Case_Study_of_Integrated_Management_of_Childhood_Illness_IMCI_Implementation_in_Kenya

[25] Fick C. Twenty years of IMCI implementation in South Africa: Accelerating impact for next decade. Johannesburg: Wits Reproductive Health and HIV Institute, University of Witwatersrand; 2017; p. 207–214.

